# An Unconventional Oral Candidiasis in an Immunocompetent Patient

**DOI:** 10.3390/jof9030295

**Published:** 2023-02-24

**Authors:** Alessandra Fusco, Maria Contaldo, Vittoria Savio, Adone Baroni, Giuseppe A. Ferraro, Dario Di Stasio, Alberta Lucchese, Adriana Chiaromonte, Giovanna Donnarumma, Rosario Serpico

**Affiliations:** 1 Department of Experimental Medicine, University of Campania “Luigi Vanvitelli”, 80138 Naples, Italy; 2 Multidisciplinary Department of Medical-Surgical and Odontostomatological Specialities, University of Campania “Luigi Vanvitelli”, 80138 Naples, Italy; 3 Unit of Dermatology, Department of Mental Health and Physics and Preventive Medicine, University of Campania “Luigi Vanvitelli”, 80100 Naples, Italy

**Keywords:** *Candida* spp., oral candidiasis, antimycogram, hyphae, biofilm, yeast cell

## Abstract

Oral candidiasis (OC) is an opportunistic fungal infection of the oral mucosae, sustained by *Candida albicans* or other non-albican *Candida* species (NAC), usually eradicated by conventional antifungals of the classes of azoles, polyenes, or derivative from echinocandins. OC usually occurs under predisposing local or systemic factors. *C. lusitaniae* is an opportunistic strain that is rarely responsible for human infection and occurs mainly in severe immunocompromised states. The present work reported an unconventional case of OC in an otherwise healthy immunocompetent woman sustained by *C. lusitaniae* and a multi-resistant strain of *C. albicans*.

## 1. Introduction

The genus *Candida* includes more than 150 species, some of which are components of the oral microbiota in 40–60% of healthy individuals and can become infectious following local or systemic alterations of the host’s defenses [1]. The incidence of opportunistic fungal infections has increased significantly in recent years, probably due to various triggers, including broad-spectrum antibiotics, immunosuppressive therapies, blood and solid-organ transplants, cancer, diabetes, and AIDS [2,3]. Moreover, it has recently been shown that there is also a rare genetic component that predisposes to susceptibility to fungal infections and that has been identified in the malfunction of NADPH-oxidase [4,5] and the abnormal production of some cytokines, such as the tumor necrosis factor α and interleukin 10 [6].

A high rate of morbidity characterizes oral candidiasis. It causes chronic pain and discomfort to the patient, especially during food intake, by interfering with chewing and swallowing [3]. Furthermore, relapses are very frequent following the interruption of antifungal therapy, which occurs in about 20% of the cases, thus persisting in 30% of them [1].

*Candida albicans* is the most represented species responsible for oropharyngeal candidiasis, but other species have also been identified at a lower incidence and only in particular host conditions. Indeed, these species lack many virulence factors owned by *C. albicans*, such as the hyphal phenotype, the ability to adhere to the oral epithelium, and high proteinase secretion. For these reasons, the infections sustained by them are usually less severe. *C. glabrata*, *C. krusei*, *C. parapsilosis*, *C. dublieniesis*, *C. tropicalis*, *C. kefyr*, and *C. guilliermondii* are the most frequently isolated species in this group, while *C. inconspicua*, *C. lusitaniae*, *C. norvegensis*, and *C. rugosa* are found less frequently [6].

In particular, *C. lusitaniae* (teleomorph *Clavispora lusitaniae*) is a rare opportunistic pathogen normally present in animal mycobiota but found only in about 1% of human adult and pediatric patients with candidemia, mainly in low-birthweight infants, immunocompromised patients, or in the course of hematologic malignancies or chemotherapy. Other risk factors are dialysis, venous catheters, neutropenia, long-term corticosteroid therapy, bone marrow transplant, and prolonged broad-spectrum antibiotics administration [7,8,9].

The peculiar characteristic of *C. lusitaniae* is the ability to possess resistance to polyene antifungal amphotericin B or to acquire it in vivo during therapy, causing breakthrough infections [7,8,9,10]; so much to be considered fatal before the era of fluconazole [7].

The present paper described a co-occurrence of oral candidiasis sustained by *C. lusitaniae* and multi-resistant strains of *C. albicans* in an immunocompetent woman.

## 2. Case Report

**Figure 1:**
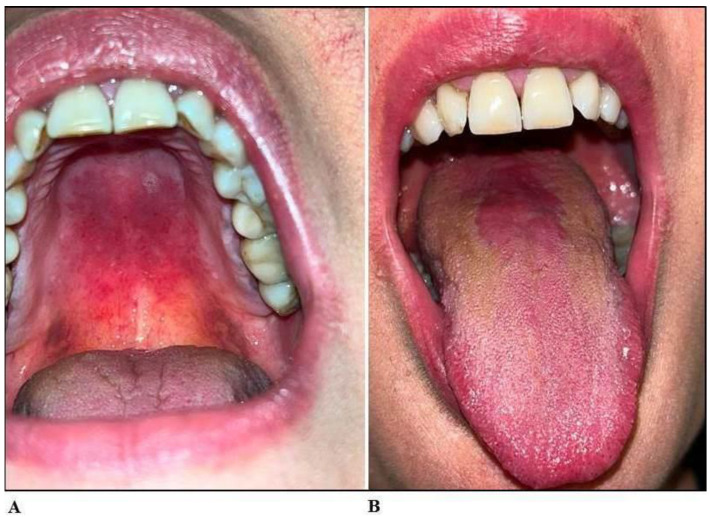
Oral Candidiasis clinical features. (**A**) Erythema and erosive areas at the palate. (**B**) Erythematous-atrophic glossitis.

On 17 February 2022, a 46-year-old woman was admitted to the Unit of Oral Pathology of the University of Campania “Luigi Vanvitelli”, Naples, Italy, because of odynophagia and burning sensation diffused to the mouth for over a year with failure to thrive despite the recurrent therapies based on topical and systemic antifungals, at empiric and not well-defined dosages and posology. At the anamnestic interview, she reported being a long-time heavy smoker (meanly 20 cigarettes per day in the last 20 years), not suffering from diabetes, and not recently subjected to antibiotic/steroid therapies, nor referring to other systemic or local diseases. She recently underwent professional tooth cleaning (scaling and root planning) and dental prophylaxis with chlorhexidine digluconate three times per day for two weeks, stopped three weeks before. At the clinical examination, she had diffused erythematous and erosive areas affecting both the hard and soft palates, while the lingual surface was partly erosive and partly atrophic, resembling the clinical picture of atrophic glossitis (Figure 1A,B).

As the clinical picture was indicative of various infective and autoimmune diseases, we asked for a series of lab tests and oral swabs for a differential diagnosis to define the etiology of such lesions (see Supplementary Materials). We hypothesized a dysimmune/autoimmune disease with fungal superinfection. Hence, since the burning symptomatology was scored on the VAS scale as “unbearable” (score = 10) by the patients, and based on the clinical signs of candidiasis, the patient was discharged with empiric therapy based on fluconazole tablets at the dosage of 100 mg for 14 days, according to clinical guidelines, as first-line approach [11]. Before dismissing the patient, we performed a further culture swab to be examined at our Unit of Virology and Microbiology of the University of Campania, “Luigi Vanvitelli”. 

The serological investigations, obtained three weeks later, on March 7, excluded autoimmune diseases, such as lupus and lupus-like syndromes, pemphigus, and pemphigoids, and the pictures were inconsistent for oral lichen planus. Furthermore, other serological values were in the normal ranges and without signs of immunodeficiency, while HIV, Herpes virus, HBV, HCV, and HAV antigens were negative. Last, there were no iron or vitamin deficiencies, coagulation disorders, or rheumatic diseases, and the urine culture was negative. The erythrocyte sedimentation rate was only slightly higher than the normal range (see Supplementary Materials).

Both culture swabs, one performed out of our structure and one at our microbiology unit lab, confirmed the diagnosis of oral candidiasis. In detail, the external lab, whose results were obtained on March 30, reported “numerous *C. abicans* colonies”, and the relative antimycogram reported sensitivity only to amphotericin B and resistance to all other common antifungals assessed. The technique used to assay the antifungals is unknown to us.

At the same time, in our lab, culture swabs were accurately obtained from the tongue, buccal mucosa, and palate and were examined on Sabouraud supplemented with chloramphenicol and gentamicin. Microbiological culture revealed creamy, rapidly growing, white colonies, and the identification performed by mass spectrometry and MALDI TOF MS confirmed candidiasis and the occurrence of two strains: *C. albicans* and *C. lusitaniae*. Our antifungal susceptibility tests, carried out by Sensititre YeastOne^®^ Systems, confirmed the resistance of *C. albicans* to all the antifungals tested (5-flucytosine, anidulafungin, caspofungin, fluconazole, itraconazole, micafungin, posaconazole, voriconazole, nystatin, and amphotericin B). Conversely, *C. lusitaniae* was sensitive to all the antifungals mentioned above except for 5-flucytosine, amphotericin B, caspofungin, and voriconazole (Table 1).

At this point, due to the finding of a multi-resistant *C. albicans* and a rare strain of *C. lusitaniae*, we requested total Ig counts and subclass lymphocyte subsets identification to establish any underestimated immune defect, which generally accompanies the appearance of candidiasis sustained by *C. lusitaniae* and also revisited the patient three weeks after the end of the antifungal treatment with fluconazole. On this occasion, on 13 April, she reported relief of symptomatology (VAS scale = 5) and an improvement of the clinical signs on the palate. However, with the median glossitis still persistent, we repeated the culture swab. This second swab reported the lack of *C. lusitaniae*, compatible with its sensitivity to fluconazole, and, as expected, the persistence of *C. albicans*, still multi-resistant to all antifungals tested. Hence, fluconazole only eradicated *C. lusitaniae*. 

The laryngoscopy performed at the ORL unit on 27 April did not reveal any pharyngeal involvement, and the additional serological tests exhibited by the patients on May 19 excluded any pathological finding related to the subclasses of Ig (IgG1–4, IgM, and IgA in the normal ranges) as well as a normal distribution, percentages, and count of the various lymphocyte’s subclasses. Meanwhile, the patient was provisionally treated with VSL#3, a commercially available probiotic containing a mixture of eight bacterial strains (*Lactobacillus acidophilus*, *Lactiplantibacillus plantarum*, *Lacticaseibacillus casei*, and *Lactobacillus delbrueckii* subsp. *bulgaricus*, *Bifidobacterium breve*, *Bifidobacterium longum*, *Bifidobacterium infantis*, and *Streptococcus salivarius* subsp. *thermophilus*) [12] at the regimen of one sachet per day for 20 days to retain in the oral cavity for 2 min and then swallowed. In addition, the patient was instructed to apply a povidone-iodine 10% solution (Betadine^®^ 10%) topically with a sterile gauze on the palate and dorsal tongue twice daily for 20 days.

A month later, on June 28, a stool culture revealed numerous *C. albicans* colonies in the feces, sensitive to all antifungals tested (amphotericin B, clotrimazole, econazole, miconazole, flucytosine, nystatin, griseofulvin, fluconazole), except for ketoconazole. 

On this basis, we performed a third culture oral swab on July 1, which reported the persistence of *C. albicans*, which was inexplicably sensitive to all the antifungals tested as the fecal strain (Table 2).

**Figure 2:**
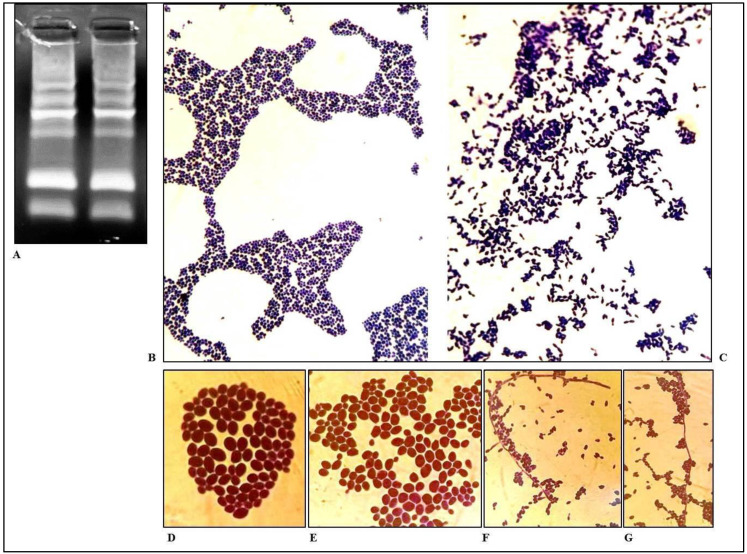
Comparison between *C. albicans* from the first and the last swab. (**A**) Genomic fingerprinting of *C. albicans* from the first and the last swabs obtained by RAPD-PCR. (**B**–**G**) Gram staining of *C. albicans* from the last (**B**) and first swabs (**C**) at 40× magnification. (**D**,**E**) Gram staining of *C. albicans* from last swab at 100× magnification. (**F**,**G**) Gram staining of *C. albicans* from first swab at 100× magnification. Note the hyphal pattern in the first swab (**C**,**F**,**G**) and the yeast cells in the last swab (**B**,**D**,**E**).

On this evidence, we hypothesized a second strain of antifungal-sensitive *C. albicans* overcoming the multi-resistant strain. Genomic DNA of both *C. albicans* multi-resistant and sensitive strains were extracted and amplified by RAPD-PCR to obtain a genomic fingerprinting to confirm this hypothesis. However, the results (Figure 2A) revealed that the two strains showed the same genomic profile, thus concluding they were the same strain.

Then, we also proceeded to analyze the morphology of *C. albicans* colonies from the first and last swabs by Gram stain and microscope observation. Examination revealed that the first culture showed a hyphal pattern, while the last showed yeast cells (Figure 2B–G).

The hyphal phenotype is typically associated with biofilm formation [13,14]. We, therefore, hypothesized that the transition from the multi-resistant to the multi-sensitive form could be associated with a biofilm/planktonic cell phenotypic switch. Hence, to confirm the ability to form biofilm of multi-resistent strain, biofilm formation assays were performed on the isolated strains from the first and last swabs using the crystal-violet staining method [15]. 

**Figure 3:**
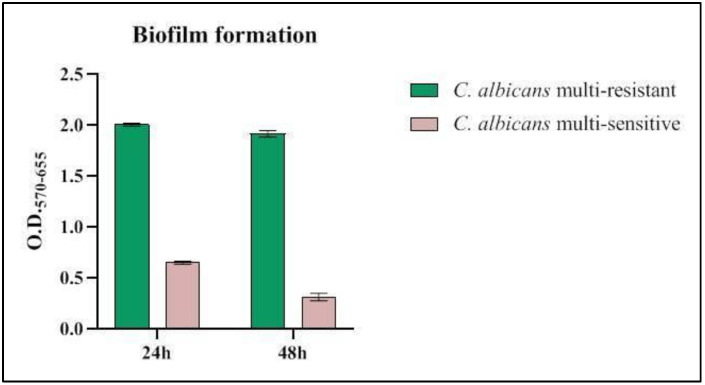
OD readings at 570–655 nm of biofilm growth of *C. albicans* from first and last swabs after 24 and 48 h.

As shown in Figure 3, the multi-resistant strain was found to form in vitro a large amounts of biofilm, which persists over time, unlike the multi-sensitive strain.

Therefore, supported by the latest antimycogram, the patient has been successfully treated with topical nystatin 100,000 IU/mL oral suspension at a regimen of 8 mL per day for two weeks.

**Figure 4:**
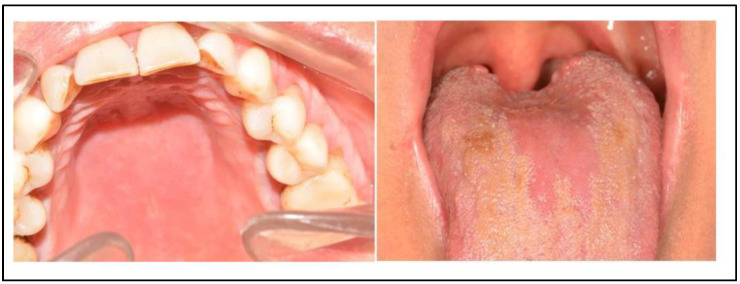
Clinical feaures of oral lesions after therapy. The brownish areas on the tongue are due to povidone-iodine pigmentation and persisted smoking.

At the scheduled control, on July 15, the referred patient ceased having a burning sensation (VAS scale = 0) and, at the clinical examination, there were no signs of inflammation of the palate or glossitis (Figure 4). A confirmatory swab was assessed, which confirmed the eradication of the infection. At the follow-up a month later, the lack of symptoms and signs persisted.

## 3. Discussion

Here we presented a clinical case of oral candidiasis sustained by a strain of *C. lusitaniae* and a strain of *C. albicans* that become chronic and multi-resistant following biofilms formation. The uniqueness of this case, first, consists of the presence in an immunocompetent patient without current or previous diseases, of a rare species, *C. lusitaniae*, which up to now was only sporadically found in immunocompromised individuals or in patients with severe pathologies. Second, the co-occurrence of a multi-resistant strain of *C. albicans* and its ability to carry out a phenotypic switch with regression of virulence factors is a further extraordinary event, as we usually witness the reverse process with the acquisition of multiple resistances.

During coinfection, the *C. albicans* strain exhibited a hyphal phenotype, typically associated with biofilm formation [13,14]. Biofilm is, by definition, represented by a complex network of polymorphic cells, including hyphal, pseudohyphal, and round yeast cells wrapped in an extracellular matrix (ECM), in which multifactorial resistance occurs, thus involving the physiological state of cells, overstimulation of drug efflux pumps, and the limitation of the diffusion of the antifungal drugs through biofilms exerted by the ECM [15].

It is known that the formation of polymicrobial biofilms from cross-kingdom interaction between *C. albicans* and oral bacteria (such as *Streptococcus mutans* and *Staphylococcus aureus*) predispose in a particular way to the onset of oral candidiasis, and that in these cases the combined administration of povidone-iodine and fluconazole is particularly effective as it is able to completely inhibit the carriage of *C. albicans* and the development and growth of the biofilm [16,17].

The therapeutic approach used in the presented case, which initially involved the administration of fluconazole and, subsequently, the intake of the probiotic mixture and the local application of povidone-iodine, probably had a beneficial effect by acting at different levels. First of all, fluconazole served to effectively eradicate *C. lusitaniae*; we cannot explain how *C. lusitaniae* occurrence was possible but we excluded occult tumors and hypothesized food contamination since the literature reported its presence in beverages, such as orange juice [18], which the patient used to drink daily.

Furthermore, we could assume that the fluconazole therapy also played a role in the reversal switch of *C. albicans* from a multi-resistant hyphal phenotype to yeast cells. In fact, it has recently been reported that fluconazole exhibits anti-biofilm activity even against resistant strains of *C. albicans* through a dual mechanism: it reduces the production of branched α-1,2 and α-1,6 mannans (WSPs), which together with β-1,6 glucans (ASPs) constitute a mannan-glucan complex (MGCx), a fundamental component of the ECM, and also interferes with the ergosterol pathway, necessary for the formation of hyphae [15], inhibiting the phenotypic switch from yeast to hypha.

On the other hand, the intake of probiotic supplements restored the normal microflora, as well as having an antimicrobial effect: it was demonstrated [19,20,21,22] that VSL#3 can induce the expression of Human-beta defensin-2 (HBD-2), an antimicrobial peptide costitutively expressed by various epithelia or induced by endogenous or exogenous stimuli and active against Gram-positive and Gram-negative bacteria, fungi and some viruses, known for its ability to inhibit the invasiveness and biofilm formation of *Candida* spp. [23,24].

Last, the literature reports the efficacy of povidone-iodine as an antiseptic for fungal mucosa infections and its inhibitory and antifungal activity, especially against *C. albicans* [25]. In detail, its efficacy was reported in relieving the symptoms of vaginal candidiasis and restoring the vaginal milieu [26,27] and in reducing the invasion of the mucosa by fungal cells [28]; it has also been proposed to prevent oropharyngeal candidiasis in pediatric bone marrow transplant patients as effective and non-toxic method [29], leading to the complete eradication of *Candida* biofilm [30].

In conclusion, all these events may have contributed to reducing the fungal biofilm formation, which is the main reason for antifungal resistances, thus allowing nystatin to work efficiently, topically—during the rinsing—and at the intestinal level—after swallowing.

To the authors’ knowledge, no similar cases have been reported by the literature so far.

## Figures and Tables

**Table 1:** Antimycogram performed after fungal identification.

Antifungals	*C. albicans*	*C. lusitaniae*
5-Flucytosine	32 NI	≤0.06 NI
Amphotericin B	>8 R	1 S
Anidulafungin	>8 R	≤0.015 S
Caspofungin	>8 R	0.06 NI
Micafungin	>8 R	0.015 S
Fluconazole	>256 R	0.25 S
Itraconazole	>16 NI	0.03 S
Posaconazole	>8 R	0.03 S
Voriconazole	>8 R	≤0.008 NI
Nystatin	>8 R	≤0.06 NI

**Table 2:** Comparison between antimycogram performed before and after first treatment with fluconazole and probiotics.

Antifungals	*C. albicans* Multi-Sensitive	*C. albicans* Multi-Resistant
5-Flucytosine	≤0.25 S	32 NI
Amphotericin B	≤1 S	>8 R
Anidulafungin	≤0.25 S	>8 R
Caspofungin	≤0.25 S	>8 R
Micafungin	≤0.25 S	>8 R
Fluconazole	0.25 S	>256 R
Itraconazole	≤0.25 S	>16 NI
Posaconazole	≤0.06 S	>8 R
Voriconazole	≤0.12 S	>8 R
Nystatin	≤0.25 S	>8 R

## Data Availability

The data presented in this study are available within the article and in Supplementary Materials.

## Supplementary Materials

The following supporting information can be downloaded at: https://www.mdpi.com/article/10.3390/jof9030295/s1, Table S1: Patient serological values.
