# Supplementary material for: An Unconventional Oral Candidiasis in an Immunocompetent Patient
Source: J Fungi (Basel). 2023 Feb 24;9(3):295. doi: 10.3390/jof9030295 (PMC10059796; doi:10.3390/jof9030295)
Supplement: Supplementary file 1 [file jof-09-00295-s001.zip › jof-2190049-supplementary.pdf]

Table S1. Patient serological values.

Variable	Patient's values	Reference range (women)
**Hematocrit (%)**	41.6	37.0–47-0
**Hemoglobin (g/dL)**	13.9	12.0–16-0
**Mean hemoglobin concentration, MHC (pg)**	32.20	27.0–31-0
**Mean corpuscolar hemoglobin concentration MCHC (g/dL)**	33.5	31.5–37.5
**White-cell count (per mm^3)**	7,970	4,000–10,000
**Differential count (%)**		
**Neutrophils**	65.90	40.00–74.00
**Lymphocytes**	27.60	19.00–48.00
**Monocytes**	5.60	0.00-12.00
**Eosinophils**	0.50	0.00–7.00
**Basophils**	0.40	0.00–2.50
**Platelet count (per mm^3)**	297,000	150,000–400,000
**Mean platelet volume (μm^3)**	9.90	7.00–11-00
**Mean corpuscular volume (μm^3)**	95.90	80–100
**Red-cell distribution width RDW (%)**	14.20	11.5–14.5
**Prothrombin time International normalized ratio INR**	0.90	1.1
**Partial Thromboplastin Time (PTT) (sec)**	28	<40
**Erythrocyte sedimentation rate (ESR) (mm/h)**	21 ↑	<15
**Fibrinogen (mg/dL)**	322	200–400
**C-reactive protein (CRP) (mg/dL)**	0.01	<6
**Complement C3 (mg/dL)**	138	82–193
**Complement C4 (mg/dL)**	26	15-57
**Rheumatoid factor (RF) IU/ml**	21	<30
**Glucose (mg/dL)**	91	70–110
**Uraemia (mg/dL)**	28	10–50
**Glutamyl oxaloacetic transaminase (GOT) U/l**	19	0–40
**Glutamyl pyruvic transaminase (GPT) U/l**	26	0–59
**Gamma-glutamyl transferase (GGT) U/l**	20	<40
**Iron (µg/dL)**	66	50–170
**Transferrin (mg/dL)**	329	200–360
**Ferritin (ng/ml)**	50.3	12–135
**Folic acid (nmol/L)**	4.9	>2.8
**Vitamin B12 pg/mL**	544	187–883
**Anti-Skin Antibody (ASA)**		
**to intercellular substance**	<1:20	<1:20
**to basement membrane**	<1:20	<1:20
**Anti-Desmoglein type 1 (Anti-DSG1) RU/mL**	<2.0	<20
**Anti-Desmoglein type 2 (Anti-DSG2) RU/mL**	<2.0	<20
**Anti-Bullous Pemphigoid 180 (BPAG180) RU/mL**	<2.0	<20
**Anti-Bullous Pemphigoid 230 (BPAG230)**	<2.0	<20

**RU/mL**		
**Antinuclear Antibodies (ANA)**	0.5	<1
**Extractable Nuclear Antigens (ENA)**		
**Ssa Ro (AU/mL)**	0.7	<12
**Ssb La (AU/mL)**	0.9	<12
**Sm (AU/mL)**	1.1	<12
**RNP (AU/mL)**	0.9	<12
**Scl 70 (AU/mL)**	1.4	<12
**JO 1 (AU/mL)**	1.3	<12
**Cenp-B (AU/mL)**	1.1	<12
**Antibodies to Herpes simplex virus type 1/2**		
**IgM (AU/mL)**	0.8	<2
**IgG (AU/mL)**	1.1	<2
**Antibodies to Hepatitis C virus (HCV)**	absent	absent
**Antibodies (Ab) and Antigens (Ag) to Hepatitis B virus (HBV)**		
**HBsAb**	2.0	low
**HBeAg**	negative	negative
**ABeAb**	absent	absent
**HBcAb IgM**	absent	absent
**Antibodies to Hepatitis A virus (HAV)**		
**IgG**	absent	absent
**IgM**	absent	absent
**Total IgE (U/mL)**	19	<150
